# Crosstalk between omega-6 oxylipins and the enteric nervous system: Implications for gut disorders?

**DOI:** 10.3389/fmed.2023.1083351

**Published:** 2023-03-28

**Authors:** Marine Mantel, Pascal Derkinderen, Kalyane Bach-Ngohou, Michel Neunlist, Malvyne Rolli-Derkinderen

**Affiliations:** ^1^Nantes Université, Inserm, The Enteric Nervous System in Gut and Brain Disorders, Nantes, France; ^2^CHU Nantes, Inserm, Nantes Université, The Enteric Nervous System in Gut and Brain Disorders, Nantes, France

**Keywords:** omega-3, omega-6, enteric nervous system, diet, inflammation

## Abstract

The enteric nervous system (ENS) continues to dazzle scientists with its ability to integrate signals, from the outside as well as from the host, to accurately regulate digestive functions. Composed of neurons and enteric glial cells, the ENS interplays with numerous neighboring cells through the reception and/or the production of several types of mediators. In particular, ENS can produce and release n-6 oxylipins. These lipid mediators, derived from arachidonic acid, play a major role in inflammatory and allergic processes, but can also regulate immune and nervous system functions. As such, the study of these n-6 oxylipins on the digestive functions, their cross talk with the ENS and their implication in pathophysiological processes is in full expansion and will be discussed in this review.

## Introduction

The gastrointestinal tract (GI) fulfils complex tasks that are essential for survival including food digestion, absorption of nutrients and transit of luminal content. These processes are coordinated by a unique peripheral autonomic nervous system embedded in the gut wall and called the enteric nervous system [ENS; (1)]. The ENS consists of ganglionated networks organized in two main plexi, myenteric and submucosal, which regulate muscle and mucosal functions, respectively. Each ganglion is composed of neurons surrounded by enteric glial cells (EGCs) and these two cell types regulate GI functions *via* specific neuro- and glio-mediators. Regarding the enteric neurons, the most common neurotransmitters are acetylcholine, vasoactive intestinal peptide and nitric oxide while, among the numerous soluble mediators produced and released by EGCs, neuroptrophic factors such as glial derived neurotrophic factor (GDNF), brain-derived neurotrophic factor (BDNF) and epidermal growth factor (EGF) have been by far the most widely studied. More recent observations showed that enteric neurons and glial cells are also capable of producing bioactive lipids such as polyunsaturated fatty acids (PUFAs) and in particular oxylipins (2, 3). Oxylipins are associated with a diverse set of inflammatory processes linked to various diseases, including inflammatory bowel diseases (IBD). The production of some n-6 pro-inflammatory oxylipins is increased in the lower GI tract of IBD patients compared to control subjects (4–7) or in inflamed mucosa relative to non-inflamed mucosa (8–11) and correlate with inflammation severity (12). These findings support the idea of an imbalance between the n-6 pro-inflammatory and n-3 anti-inflammatory derivatives as pathological contributing factors, but this concept was challenged when it has been found for several derivatives that they could have pro- as well as anti-inflammatory properties (13–18). More importantly, recent findings define the importance of pro-resolutive properties of some n-6 derivatives (19) and their homeostatic functions (20, 21). In addition, we recently reported that EGCs produce mostly n-6 and little n-3 oxylipins and that some n-6 oxylipins that have a pro-resolutive and pro-homeostatic function are lacking in Crohn’s disease patients. The general effects of n-6 oxylipins on the GI tract have been reviewed elsewhere (22, 23) but as far as we know there is no existing review on the links between n-6 oxylipins and the ENS. The current article fills that void and covers the research in this subject area over the past two decades. Here, we first provide a brief overview of n-6 oxylipins, before discussing the interplay between the compound and the ENS and their potential roles in GI pathophysiology.

## n-6 oxylipins

**Figure 1:**
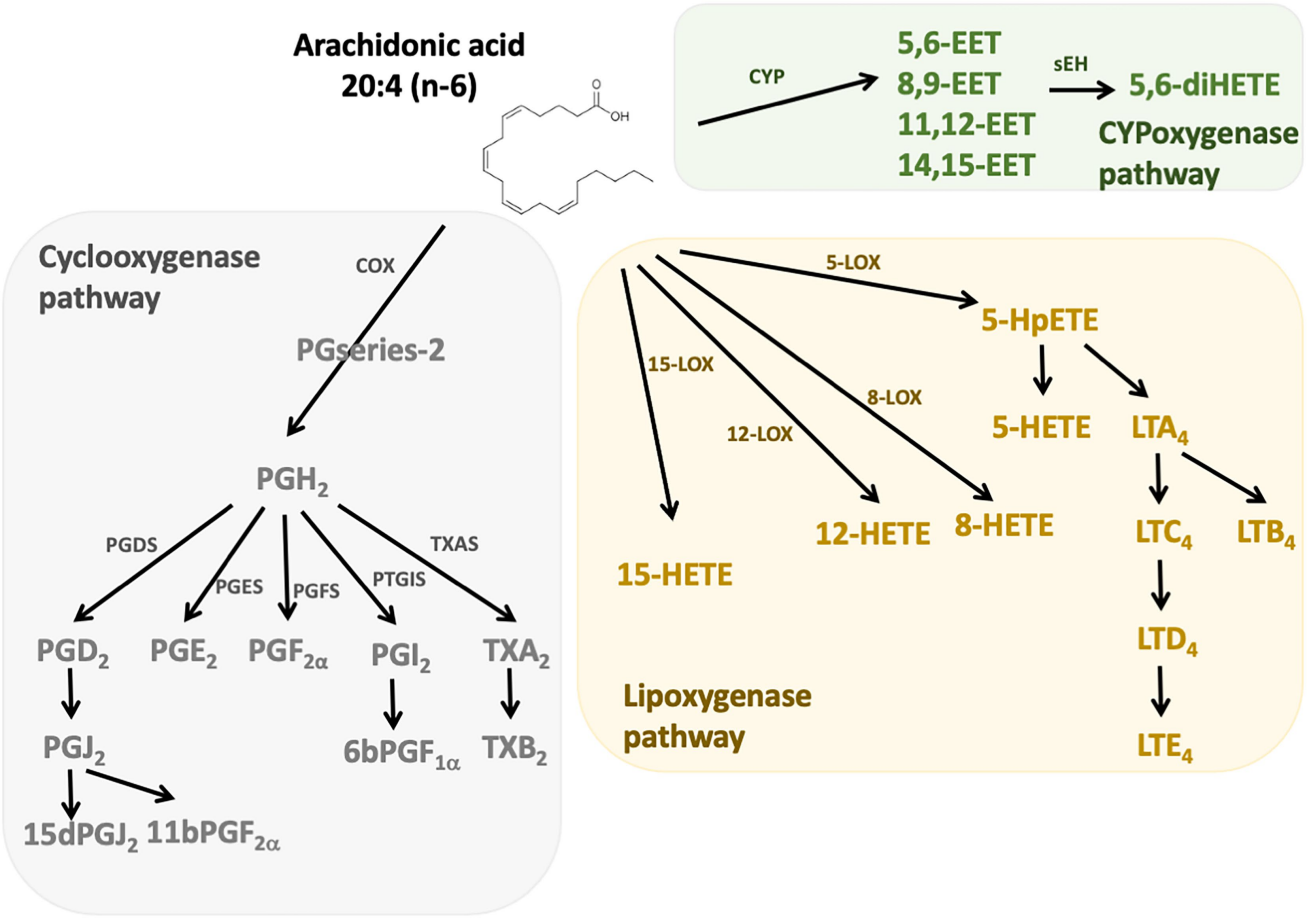
n-6 Oxylipins derived from arachidonic acid. The metabolism of PUFAs is a complex process involving several enzymes of desaturation, elongation, and β-oxidation. n-6 oxylipins are synthesized from arachidonic acid (ARA) through three major pathways, the first depending on cyclooxygenase (grey), the second on lipoxygenase (yellow), and the third on cytochrome P450 (CYP) monooxygenase activities (green). COX, cyclooxygenase; LOX, lipoxygenase; CYP, cytochrome P450; PGFS, prostaglandin F synthase; PGDS, prostaglandin D synthase, PGES, prostaglandin E synthase; PTGIS, prostaglandin I synthase; TXAS, thromboxane A2 synthase.

The time is long gone when lipids represented only inert fats. In addition to their role in the formation of biological structures (e.g., cell membranes or organelles), lipids have metabolic energy storage and cell signaling function. Signaling lipids, from lysophosphatidic acid to endocannabinoids, regulate essential functions such as synaptic transmission, vasoconstriction or inflammation. Among the most important are the Omega-3 (n-3) and Omega-6 (n-6) oxylipins, the family of oxygenated products formed from n-3 and n-6 PUFAs. In both Omega families, many forms of PUFAs exist: *α*-linolenic acid (ALA), eicosapentaenoic acid (EPA), and docosahexaenoic acid (DHA) from the n-3 family and linoleic acid (LA), dihomo-*γ*-linolenic acid (DGLA), and arachidonic acid (ARA) from the n-6 family are the important PUFAs for human health.

n-6 Oxylipins are synthesized from ARA by cyclooxygenase, lipoxygenase, and cytochrome P450 (CYP) monooxygenase activities (24; Figure 1). Cyclooxygenases (COX), also known as prostaglandin-endoperoxide synthase (PTGS) are oxidoreductases that allow the synthesis of prostaglandins (PG) and thromboxanes (TX). In humans there are two isoforms of COX: COX-1 and COX-2. COX-1 participates in the basal synthesis of eicosanoids and is constitutively expressed in most tissues. In contrast, the expression of COX-2 is induced by stimuli such as inflammation (25). Their actions are carried out in two stages: first they subtract a hydrogen from the carbon 13 of the ARA and they add two molecules of oxygen, which allows the formation of prostaglandin G_2_ (PGG_2_). Second, this PGG_2_ is reduced to PGH_2_ by active peroxidase. The synthesis of PG and TX is then carried out thanks to specific isomerases. Thus, the PGD synthetases (L-PGDS and H-PGDS), the PGE synthetases (mPGES-1, mPGES-2, and cPGES3), the PGF synthetase (PGFS), the PGI synthetase (PTGIS and the TX synthetase (TXAS) that allow the synthesis of PGD_2_ (which can give PGJ_2_ following dehydration), PGE_2_, PGF_2α_, PGI_2_ (or prostacyclin), and TXA_2_, respectively. The receptors for these compounds are G-protein coupled receptors, and a same eicosanoid can modulate its action depending on the targeted receptor subtype (26). The lipoxygenase (LOX) pathway allows the synthesis of hydroxyeicosatetraenoic acids (HETE) and leukotrienes (LT). These are non-heme iron enzymes that contain dioxygenases and are constitutively expressed in plants and animals. In the intestine we find the lipoxygenases 15-LOX (15-LOX-1 and 15-LOX-2), 12-LOX and 5-LOX which allow the synthesis of 15-HETE, 12-HETE, 5-HETE and LT, respectively. They will catalyze the reaction PUFA (ARA for example) + O2^–^ > Hydroperoxyeicosatetraenoic acid (HpETE). These derivatives are then transformed into more stable one: HETE and LT (27). The LT receptors are well described, but little is known about those for HETE. However, it is recognized that they may be peroxisome proliferator-activated receptor (PPAR) agonists (28, 29). On the other hand, epoxy-PUFA are the oxylipins generated by CYP enzymes, later converted to their corresponding 1,2-diols by soluble epoxy hydrolase.

## n-6 Oxylipins regulate ENS

Oxylipins play a major role in the regulation of inflammation not only in the central nervous system (CNS) but also in the GI tract. This prompted research on their potential effects on the ENS and numerous studies showed that n-6 oxylipins are indeed able to modulate neuronal activity and to exert neuroprotective effects. Regarding EGCs, although the data are still scarce, their phenotype and activity could be modified by oxylipins.

### n-6 Oxylipins, enteric neuronal activity and neuroprotection

**Figure 2:**
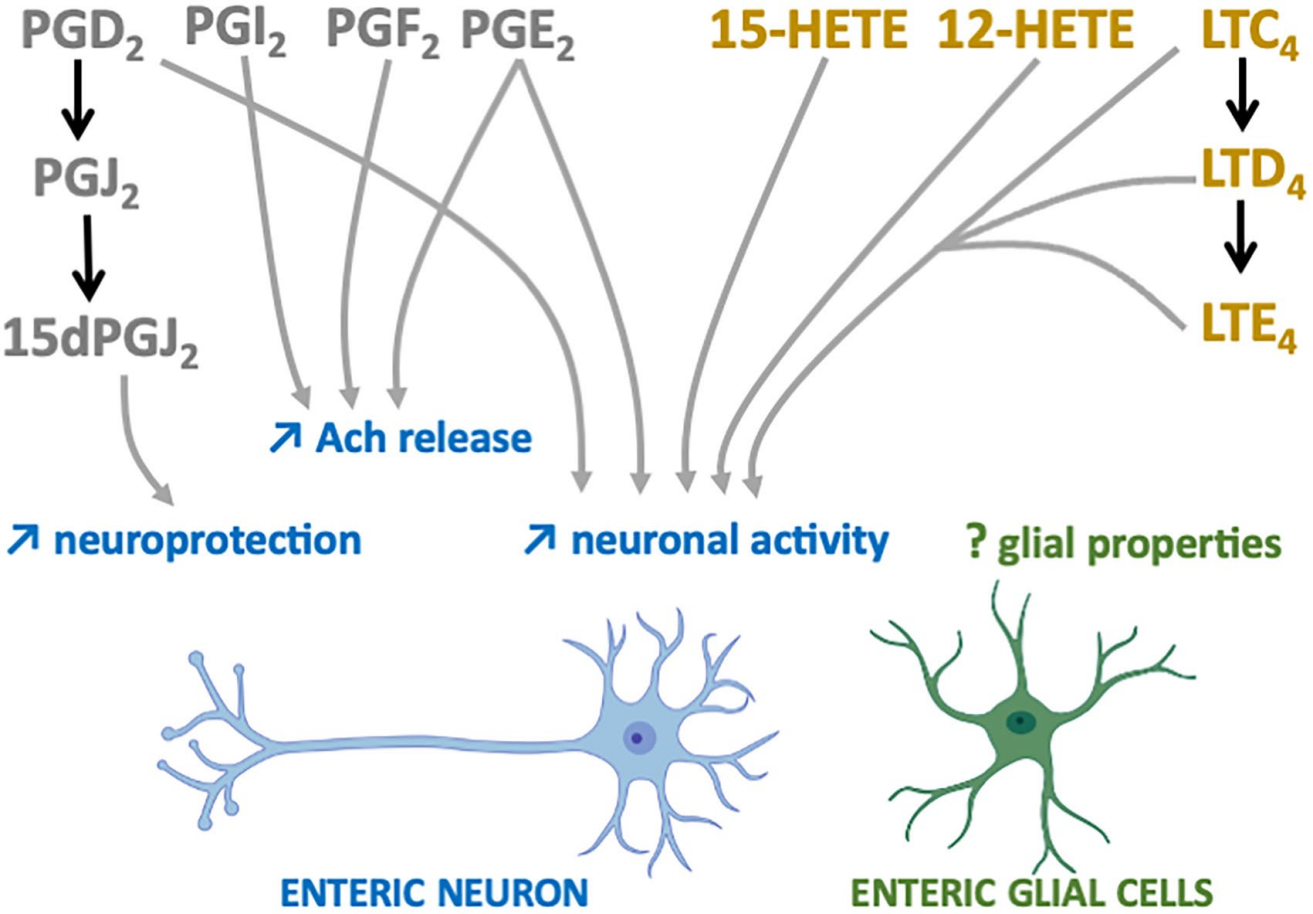
n-6 Oxylipins regulate the Enteric nervous system. COX-(grey) or LOX-(yellow) derived n-6 oxylipins increase (↑) different enteric neuron properties (blue) but their impacts on EGCs remain to be determined (?).

The first studies suggesting that n-6 oxylipins could modulate enteric neurotransmission and thus neuronal activity were performed in the myenteric plexus of guinea pig ileum and focused on the release of acethylcholine (Ach). Two independent groups showed that the release of Ach by either protein kinase C, DMPP (a nicotinic agonist) or neuronal activity was decreased in the presence of mepacrine, a PLA_2_ inhibitor, and that the opposite was observed when samples of myenteric plexus were treated with either a PLA_2_ activator (melittin) or ARA (30, 31). Additional pharmacological experiments allowed to identify the prostaglandins PGE_2_, PGI_2_ and PGF_2_ as critically involved in the chemically or electrically-induced release of Ach by enteric neurons (32–35) (Figure 2). Regarding the LOX pathway, AA861, a selective inhibitor of 5-lipoxygenase, inhibited acetylcholine release from the myenteric plexus induced by electrical field stimulation, an effect that was reversed by metabolites from the 5-lipoxygenase pathway, including 5-HETE, LT C_4_, D_4_ and E_4_ (36) (Figure 2). Besides Ach, it has also been shown that prostanoid signaling, including PGE_2_, regulated the cytokine-induced suppression of norepinephrine release in rat myenteric plexus (37). Although scarcer than for the myenteric plexus, some findings suggest that the n-6 oxylipins produced by the submucosal plexus might be also involved in the release of Ach. Interleukin-1β (IL-1β) potentiated the release of Ach induced electrical transmural stimulation in whole guinea pig ileum but not in longitudinal muscle/myenteric plexus (LMMP) only or in mucosal-free preparations and these effects were prevented with of PLA_2_, COX or LOX inhibitors (38) (Figure 2). Taken as a whole, these findings suggest that IL-1β stimulates cholinergic enteric neurons *via* a cascade of ARA metabolites produced in tissues at the mucosal and submucosal level, but not in the myenteric plexus or longitudinal muscles.

Prostaglandins are also capable of directly modulating the electrophysiological properties of enteric neurons. Treatment of myenteric ganglia from the guinea pig colon or ileum with PGE_2_ led to prolonged depolarization of enteric neurons with enhanced spike discharge together with an increased neuronal excitability (39–41) (Figure 2). More recently, myenteric neuron activity and contraction have been shown to depend on prostaglandin release (COX-2 pathway) from mucosal mast cells in a model of TNBS-induced colitis (42). Similar findings were observed when submucosal neurons of guinea pig colon were treated with PGD_2_ (43). The application of leukotrienes C_4_, D_4_ and E_4_ to myenteric and submucosal neurons of guinea pig resulted in slow depolarization of small intestine neurons, thereby suggesting that derivatives of the LOX pathway are also modulators of enteric neuronal excitability (Figure 2).

Oxylipins are well-known neuroprotectors in the CNS and this might be also the case in the ENS. 15-Deoxy-Delta-12,14-prostaglandin J_2_ (15d-PGJ_2_), a PGD_2_ metabolite produced by EGCs, has been identified as having neuroprotective effects (44) (Figure 2). Pretreatment of primary cultures of ENS with this n-6 oxylipin prevented the neurotoxicity induced by hydrogen peroxide. The involvement of EGCs in this beneficial effect was validated after glial genetic invalidation of the key enzyme involved in the synthesis of 15dPGJ_2_, the L-PGDS. The neuroprotective effect of this glial mediator appeared to be due to increased expression in enteric neurons of both glutamate cysteine ligase and intracellular glutathione.

### n-6 Oxylipins and EGCs

In sharp contrast with the regulation of enterics neuron properties described below, data on the effects of n-6 oxylipins on the EGCs are lacking. The observation showing that a high fat diet, which controls in a major way the GI oxylipin content, increased the number of EGCs in myenteric plexus in a juvenile mouse model (45), but alter submucosal glial density where the expression of the glial marker calcium-binding protein S100β (S100β) was also decreased (46), might suggest that EGC morphology and/or reactivity could be modified *via* oxylipins. But this is the few we know, and while many studies described the regulation of central glia (astrocytes or microglia) through oxylipin (mostly n-3 oxylipin), the impact of a specific oxylipin on EGC markers and functions remain to be addressed.

## The ENS is a source of n-6 oxylipins

While n-6 oxylipins can regulate the ENS, we could wonder whether ENS itself could be a source of n-6 oxylipins. Unlike other signaling, lipid messengers are produced on demand and degraded by metabolic enzymes to control their lifespan and signaling actions. The ENS contains the enzymatic machinery required for the production of n-6 oxylipins, and production of several have been measured in ENS or EGC in culture.

Secreted PLA2 (sPLA2-X) has been detected in the myenteric ganglia of small intestine and in neuronal fibers of the stomach, thereby suggesting that ARA-derived eicosanoids are produced by enteric neurons (47, 48). Regarding the cyclooxygenase pathway, both COX1 and 2 enzymes are found in the myenteric ganglia in both humans and mice (49–51). We further showed that both neurons and EGC from primary culture of rat ENS express the L-PGDS (52), and human EGC also express the aldo-keto reductase family 1 member C3 (AKR1C3) (53). The expression of these key enzymes is associated with the production of PGD_2_, 15dPGJ_2_, 11βPGF_2α_, PGE_2_, PGI_2_ (estimated by the measurement of its metabolite 6kPGF_1α_) and TXB_2_ in primary culture of rat ENS, rat or human EGC (52–54). The LOX pathway is also active in ENS as we have shown that human submucosal plexus and human EGC express the 15-LOX-2 (54). The downstream mediator, 15-HETE, or oxylipins produced from other LOX pathways such as LTB_4_, 5-HETE, 8-HETE and 12-HETE are also produced by rat and human EGC as demonstrated by the enlarged lipidomic analyzes (53, 54). No enzyme or specific metabolite from the CYP pathway has been specifically attributed to the ENS so far.

In an interesting way and comparable to what has been done in the CNS, the production of n-6 oxylipins by the ENS is regulated by inflammation. Seminal papers on the topic showed that COX2 and its downstream metabolites TXA_2_ and TXB_2_ were upregulated by TNF-α in isolated rat myenteric ganglia (55), while LPS induced COX-2 expression in enteric glial cells *via* autocrinal secretion of IL-1β (56). More recently, we showed that both neurons and EGC responded to LPS by increasing L-PGDS expression and its downstream metabolite PGD_2_ (10).

Therefore, it appears that ENS, in the same way as immune cells, muscle cells or epithelial cells, could be a source of n-6 oxylipins, and thereby regulate its neighboring cells to participate in the control of digestive functions.

## n-6 Oxylipins: ENS crosstalk and intestinal functions

**Figure 3:**
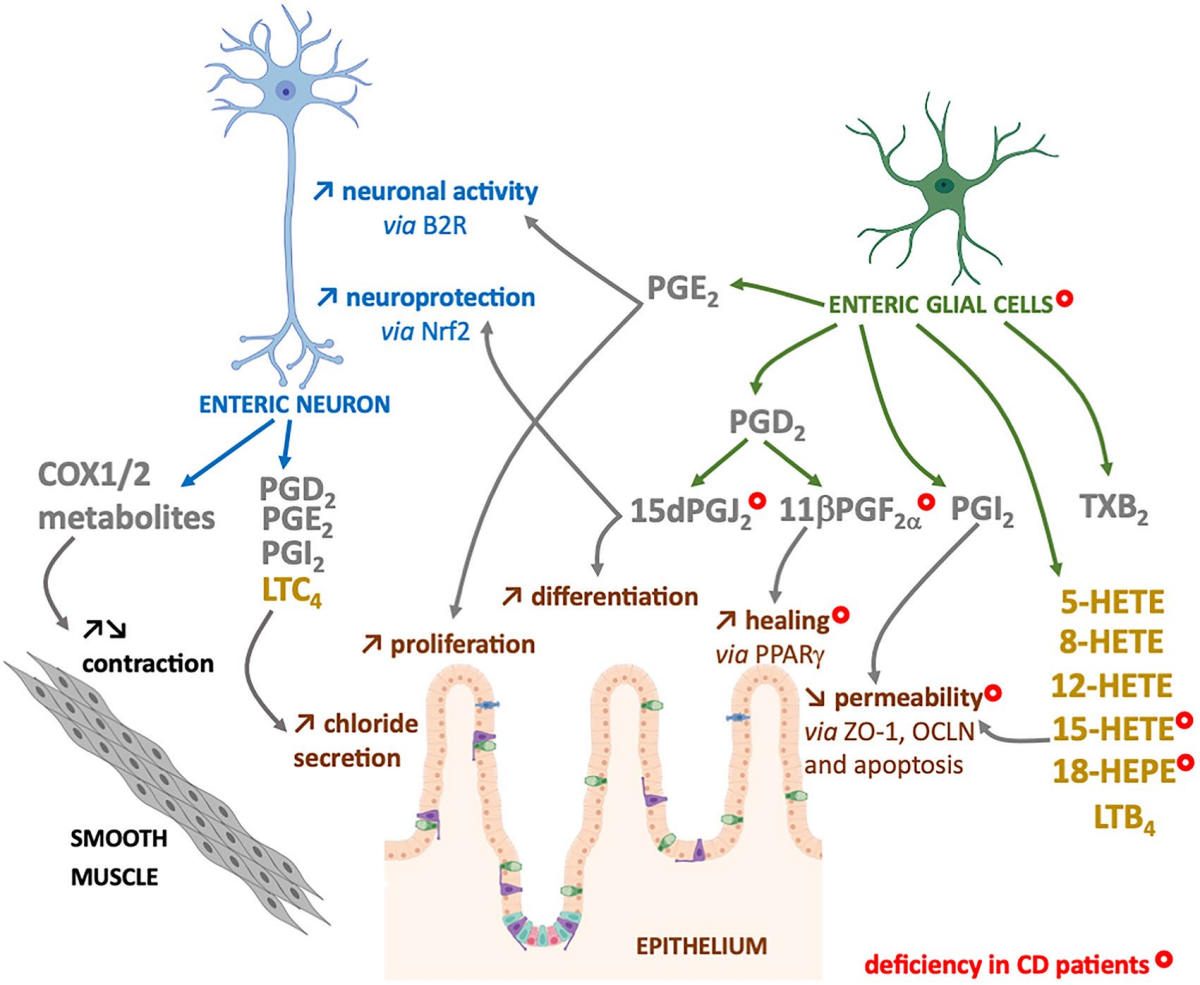
Enteric neurons and glial cells produce n-6 oxylipins to regulate the neighboring cells. Some n-6 oxylipin are to regulate glia or neurons, but also to control functions of smooth muscle or intestinal epithelial cells. COX-(grey) or LOX-(yellow) derived n-6 oxylipins produced by enteric neurons and/or glial cells can regulate different enteric neuron properties (blue), but also intestinal epithelial barrier properties (brown) or smooth muscle contraction (black). Marked with a (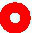) are cells, oxylipins or cellular functions deficient in chronic inflammatory disease that is Crohn’s disease (CD).

The n-6 oxylipins can be produced by enteric nervous cells, both at the neuronal and glial level, but these lipid messengers regulate ENS properties, suggesting that they could also play a crucial role in the regulation of intestinal functions controlled by ENS (Figure 3).

### Intestinal epithelial regulation

The intestinal epithelial barrier (IEB) represents a central actor of intestinal homeostasis. Intestinal epithelial barrier integrity is ensured by a constant renewal thanks to a balance between proliferation, differentiation, migration and elimination of cells, but also by a tight regulation of permeability and repair. The role of EGCs in controlling the properties of the IEB is quite well studied, and our laboratory has highlighted the crucial role of n-6 oxylipins in this regulation. Thus, the 15dPGJ_2_ induced differentiation and inhibited the proliferation of epithelial cells (52) (Figure 3). We also demonstrated the involvement of glial 11βPGF_2α_ in the control of IEB repair *via* an increase in intestinal epithelial cell spreading (53) (Figure 3). Finally, we have demonstrated that another glial mediator, the 15-HETE, controls IEB paracellular permeability, both *in vitro* and *in vivo* (54) (Figure 3). Thus, EGCs represent a source of n-6 oxylipins responsible for IEB strengthening.

### Motility regulation

Inhibitors of both COX isoforms increased the contractions of human longitudinal muscles induced by electrical stimulation and are atropine-sensitive. It has been suggested that cholinergic neurons are thought to be primarily modulated by COX-1 activity, whereas COX-2 acts primarily at the muscular level to decrease muscarinic responses (50). Another study on rat duodenum also showed that inhibition of COX isoforms with indomethacin increased motility, probably *via* enteric nerve excitation (57). While the expression of COX1 and COX2 enzymes in neurons of myenteric ganglia of mouse or human colon, their role in the neuromuscular function is unclear. The inhibition of COX does not have the same effects on neuromuscular functions (peristalsis, spontaneous or Ach-induced activity, electrically-induced contractions, etc.…) depending on the species, but also according to the intestinal region (stomach, jejunum, distal colon, etc.) or the studied muscle layer (circular/longitudinal) (58). In addition, as enteric neurons can be the target but also the source of COX-pathway metabolites, and as many studies used COX inhibition or deletion without neuronal specification, it is difficult to conclude about the specific neuronal contribution (58) (Figure 3).

The LOX pathway has also been involved in motility regulation as a recent work demonstrate how the 12-HETE, can remodel the ENS and inhibit duodenal contraction (59).

### Secretory regulation

The prostaglandins PGD_2_, E_2_, I_2_ and F_2a_ as well as LTC_4_ increased short-circuit current, which represents chloride secretion and activated submucosal enteric neurons. The secretory response was reduced by treatment with tetrodotoxin or atropine, suggesting an involvement of cholinergic submucosal neurons in this regulation (60–62). Nevertheless, the prostaglandin regulation of secretion through ENS could be more complex, being accelerated with high concentrations of PGE_2_ (63) or sometimes presenting anti-secretory effect for rat colonic preparations treated with PGD_2_ (64, 65).

### Immune system regulation

Prostanoids and leukotrienes are mediator of communication between macrophages or mast cells and ENS, but the ENS to immune cell communication through n-6 oxylipins has not been described yet.

One study demonstrated that in a co-culture of mast cells with rat submucosal neurons, inhibition of 5-LOX, resulted in a reduction of the number of responding neurons (66). In the same way, in small intestine of guinea pig sensitized to cow’s milk and exposed to beta-lactoglobulin, was observed an increase in neuronal excitability, both suppressed by treatment with a COX or 5-LOX inhibitors (67). Finally, one study suggests the involvement of macrophage-like cells found in rat myenteric plexuses in the effect of IL1β and IL-6 on the suppression of prostanoid-mediated norepinephrine release (37).

## n-6 Oxylipins and ENS dysfunctions in gut disorders

In parallel to the involvement of the crosstalk between n-6 oxylipin and the ENS in the maintenance of intestinal homeostasis, increasing evidence suggest their dysregulation and role in different pathological conditions of the gut. Although numerous pre-clinical and clinical studies showed controversial results concerning the use of oxylipins or PUFAs, there is still much more to discover about the beneficial effects of these molecules, particularly in the field of IBD (68).

### Inflammatory bowel diseases

The role of oxylipins in inflammatory bowel processes is multifaceted and unclear. Many studies associate n-6 with proinflammatory effects, and thereby an increased risk of developing IBD (8, 69–71). Conversely, many derivatives of ARA could also play an anti-inflammatory and pro-resolutive role (72), and treatment with COX inhibitors led to an exacerbation of the activity of the disease or induce relapse in IBD patients (73, 74). More recently, two eicosanoid synthesis pathway enzymes (PTGIS and PGD_2_ synthase) were identified among genes dysregulated in fibrotic Crohn’s disease (75). In the same manner, the concentration of a cluster of 10 eicosanoids (PGF2_a_, 15-HETE, TxB_2_, 11β PGF2_α_, PGE_3_, 15dPGJ_2_, 6kPGF_1α_, PGE_2_, PGD_2_ and 8 isoPGA_2_) were decreased in biospie supernatant form UC or CD patients, when biopsies came from a non-inflamed areas (76). These studies suggest that, while upregulated by acute inflammation, the defects in expression of synthesis enzyme and/or the low production of n-6 oxylipins are associated with chronic inflammation.

These changes of n-6 oxylipins production could be related to alterations of EGCs and enteric neurons observed in IBD patients. Indeed, in addition to neuronal structural alterations (77), increase in neuronal degeneration (78–81), and changes in neurochemical coding (82–85), neurons of the myenteric plexuses from active IBD patients expressed high level of COX2 that could contribute to the dysmotility observed in patients (86). Indeed, a decrease in the number of myenteric neurons per ganglion was observed after the induction colitis with TNBS in guinea pigs and especially an increase in COX2 expression and release of PGE_2_, TXA_2_ and LTB_4_ in the neuromuscular layer, was associated with a decrease in colonic propulsion and an increase in the excitability of myenteric neurons (87). In addition to neurons, EGCs from IBD patients present structural alterations (80), changes in glial marker expression (77, 88, 89) and a decrease in the production of four glial lipid mediators: the 15-HETE, 18-HEPE, 15dPGJ_2_ and 11βPGF_2α_ (53) (Figure 3). The defect in 15dPGJ_2_ and 11βPGF_2α_ production was explained by a decrease in L-PGDS and AKR1C3 synthesis enzyme expression by EGCs from CD patients (53). The functional consequences of the decrease of 15-HETE or 11βPGF_2α_ productions by CD EGC have been identified as a loss of control of the IEB permeability or healing, respectively (53, 54) (Figure 3). Similarly, the decrease of 15dPGJ_2_ could be linked to a loss of neuroprotection from oxidative stress (44) and a defect in the proliferation and differentiation of intestinal epithelial cells (52) (Figure 3).

Thus, while most of the studies are focused on an active role of n-6 oxylipins in the deleterious effect of intestinal inflammation, we have to reconsider their necessity in the resolution of inflammation (90, 91) and the possible involvement of their defects in the ENS as a contributing factor to the pathological mechanisms of IBD.

### Infectious diseases

Other studies suggest a detrimental role of n-6 oxylipins that target the ENS in several infectious diseases, especially during parasitic infections.

The *Trypanosoma cruzi* infection leads to myenteric neuron cell death mainly in the esophagus and colon and results in megaesophagus and megacolon, respectively (92). A treatment with low-dose aspirin during the acute phase of infection, known to inhibit COX, reduced parasitemia and protects myenteric neurons from cell death and plastic changes, notably their hypertrophy. This protection of cholinergic neurons was also associated with a recovered transit time by the aspirin treatment. In addition, treatment with aspirin decreases substance P and increases vasoactive intestinal peptide (VIP) productions, and could therefore help to regulate inflammation in these animals.

The infection with the parasite *Cryptosporidium* in piglet, induces intestinal villous damages associated with malabsorption and reduced sodium chloride (NaCl) absorption due to prostaglandin released from the inflamed tissue. The prostaglandins alter NaCl transport in this infection primarily by stimulating cholinergic interneurons that innervate VIPergic and cholinergic motor nerves (93). The ENS may be a potential target for pharmacological control of the acute diarrhea in this infection.

### Other intestinal disorders

The involvement of n-6 oxylipins has also been suggested in many other pathologies associated with intestinal disorders, particularly *via* their role on neuronal excitation or their inhibitory role on intestinal contractility.

Patients with irritable bowel syndrome (IBS) are characterized by abdominal cramps, diarrhea or constipation. Treatments with the PGD_2_ antagonist BW A868C inhibited the longitudinal muscle contractions potentiated by supernatant of biopsies from IBS patient (94). In addition to this effect on motility, lipidomic studies have demonstrated that n-6 oxylipins can participate in visceral sensitivity. Quantification of the PUFA metabolites produced in biopsies from control and IBS patients have shown that 5,6-EET and PGE_2_ production are increased in diarrhea-predominant IBS patients and 5-oxoeicosatetraenoic acid (5-oxoETE) in IBS with predominant constipation (95, 96). These productions are correlated with pain and bloating and these oxylipins can activate afferent sensory neurons but the contribution of ENS in these processes is not described.

In the same way, about half of the patients with slow-transit constipation exhibits an elongated colon and outlet obstruction. One study showed increased levels of COX2 in muscularis and mucosa of partially obstructed mice, associated with colon elongation, slowing of transit, and loss of mucosal reflexes. The expression of COX2 was also found in subpopulations of nNos myenteric neurons, and PGE_2_ application completely abolished spontaneous inhibitory junction potentials and depolarize the circular muscle, suggesting that it was removing tonic inhibition. Removal of the obstruction or treatment with a selective COX2 inhibitor restored control parameters as spontaneous inhibitory junction potentials and mucosal reflexes (97). This study suggested that an increase in COX2 expression led to a hyperexcitable colon, largely due to suppression of inhibitory nerve pathways by prostaglandins.

In the case of food allergies, products of activated mast cells, including prostanoids and leukotrienes, are also known to stimulate nerve endings, to modulate neuronal excitability and to increase synaptic transmission, resulting in phenotypically and functionally altered submucosal secretomotor neurons (98).

In the context of postoperative ileus, a study showed, in the rat, that a bowel manipulation increased the expression of COX2, associated with an increased PGE_2_ release in the immune cells but also in subpopulation of myenteric neurons. This associated with a decrease in contractile smooth muscle activity (99). COX2 metabolites such as PGE_2_ produced by the ENS appear to be involved in enteric dysmotility, and administration of specific inhibitors could help to prevent the transit delay induced by bowel manipulation (58). The PGE_2_ produced by the ENS is not only involved in smooth muscle control, but also in epithelial proliferation, and a study nicely shown how tumor cells can induce PGE_2_ production by EGCs to activate colon cancer stem cells and stimulate tumorigenesis (100).

Finally, some ARA derivatives could also have an emetogenic role, and one study investigated the role of leukotrienes in this effect. Injections of different leukotrienes, such as LTC_4_ and D_4_ induced an increase in the frequency and percentage of least shrews receiving vomiting. Following LTC_4_-induced vomiting, they showed increased labeling of nuclei for Fos in ENS, suggesting that leukotrienes modulate emetic activity in part *via* enteric nervous components activation (101).

## Conclusion

Nowadays, the crosstalk between n-6 oxylipins and the ENS is mainly represented by n-6 oxylipins regulation of neuronal activity and the subsequent modulation of muscle contraction Nevertheless, first evidence shows that n-6 oxylipins could also have a broader impact through their production by enteric glia to the control of the intestinal epithelial properties. The EGC-derived n-6 oxylipins that strengthen the intestinal epithelial barrier and participate in gut homeostasis, are lacking in patients with Crohn’s disease. We could speculate that they could also regulate other EGC neighboring cells such as ILC3, macrophages or T cells, and therefore could more broadly participate in pathological processes. New studies must go beyond the conventional wisdom of inflammatory prostaglandins, bring a better understanding of the kinetics, receptor type and cellular mechanisms regulated by specific n-6 oxylipins in order to elaborate new anti-inflammatory strategies, for example *via* supplementation with pro-resolutive mediators. Pharmacological approaches coupled with tissue-specific gene invalidation might facilitate the translation of fundamental discoveries into clinical solutions for gut diseases with aberrant lipid signaling.

## Author contributions

MR-D and PD directed, constructed and produced the final version of the review. MM wrote the first draft. MR-D produced the data. KB-N conducted a critical reading. MN participated in the reflexion and work funding. All authors contributed to the article and approved the submitted version.

## Funding

This work was supported by the “Institut national de la santé et de la recherche médicale (Inserm),” the Nantes Université, the SantéDige Foundation and the cluster IBD NExt from Nantes Excellence Trajectory (NExT Health and Engineering). MM benefits a doctoral fellowship managed by Bba Milk Valley, dairy industrial association. We are grateful to Regional Councils of BRetagne (grant no. 19008213) and Pays de la Loire (grant no. 2019-013227) for their financial support through the interregional project PROLIFIC. MR-D is supported by the Centre National pour la Recherche Scientifique (CNRS).

## Conflict of interest

The authors declare that the research was conducted in the absence of any commercial or financial relationships that could be construed as a potential conflict of interest.

## Publisher’s note

All claims expressed in this article are solely those of the authors and do not necessarily represent those of their affiliated organizations, or those of the publisher, the editors and the reviewers. Any product that may be evaluated in this article, or claim that may be made by its manufacturer, is not guaranteed or endorsed by the publisher.

## Abbreviations

15d-PGJ2: 15-Deoxy-Delta-12,14-prostaglandin J2
Ach: acethylcholine
AKR1C3: aldo-keto reductase family 1 member C3
ARA: arachidonic acid
BDNF: brain-derived neurotrophic factor
S100b: calcium-binding protein S100b
CNS: central nervous system
CD: Crohn’s disease
COX: cyclooxygenases
CYP: cytochrome P450
DGLA: dihomo-γ-linolenic acid
DHA: docosahexaenoic acid
EPA: eicosapentaenoic acid
EGCs: enteric glial cells
ENS: enteric nervous system
EETs: epoxyeicosatrienoic acids
EGF: epidermal growth factor
GI: gastrointestinal tract
GDNF: glial derived neurotrophic factor
GFAP: glial fibrillary acidic protein
HpETE: hydroperoxyeicosatetraenoic acid
HETE: hydroxyeicosatetraenoic acids
IBD: inflammatory bowel diseases
IL-1b: Interleukin-1β
IBS: irritable bowel syndrome
LT: leukotrienes
LA: linoleic acid
LOX: lipoxygenase
LMMP: longitudinal muscle/myenteric plexus
oxoETE: oxoeicosatetraenoic acid
PPAR: peroxisome proliferator-activated receptor
PG: prostaglandin
PGDS: PGD synthetase
PGES: PGE synthetase
PGFS: PGF synthetase
PTGIS: PGI synthetase
PLA2: phospholipase A2
PUFAs: polyunsaturated fatty acids
PTGS: prostaglandin-endoperoxide synthase
PG: prostaglandins
NaCl: sodium chloride
TX: thromboxanes
TXAS: TX synthetase
UC: ulcerative colitis
VIP: vasoactive intestinal peptide
ALA: α-linolenic acid
